# Supplementary material for: Microparticles of Sericin-Dextran Conjugate for Improving the Solubility of Antiviral Drug
Source: J Funct Biomater. 2023 May 24;14(6):292. doi: 10.3390/jfb14060292 (PMC10299130; doi:10.3390/jfb14060292)
Supplement: Supplementary file 1 [file jfb-14-00292-s001.zip › jfb-2401870-supplementary.pdf]

Microparticles of Sericin-dextran conjugate for improving the solubility of antiviral drug

Shuqi Chen,XiaolongFeng,Xinwei Life,Miaochang Liu,WenxiaGao*, Qian Miao*, Huayue Wu*

College of Chemistry and Materials Engineering, Wenzhou University, Wenzhou 325027, China

* Correspondence: wenxiag@wzu.edu.cn (W.G.); miaoqian@wzu.edu.cn (Q.M.); huayuewu@wzu.edu.cn (H.W.); Tel./Fax: +86-577-88368280 (H.W.)

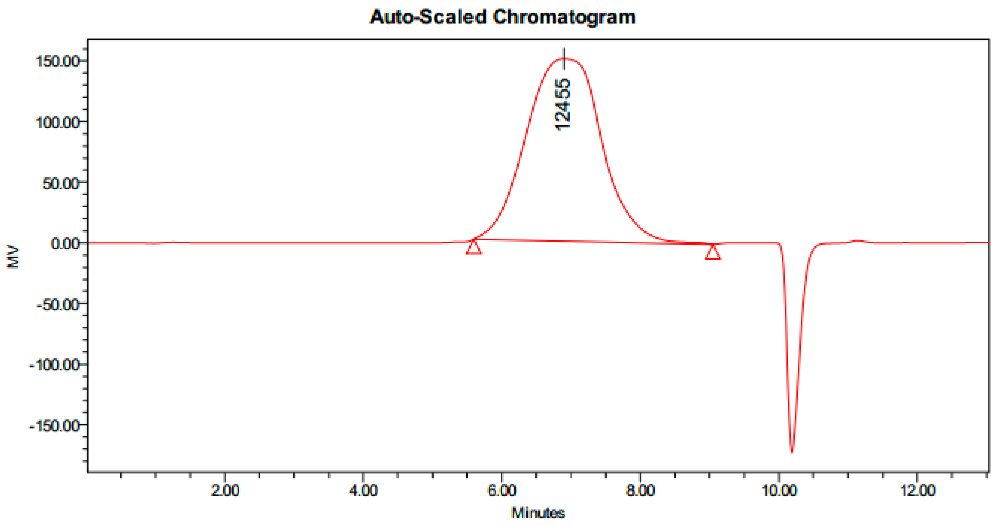

GPC Results

	Retention Time (min)	Area (磺*sec)	% Height	% Area	Mn	Mw	MP	Mz	Mz+1	Polydispersity
1	6.896	11398883	100.00	100.00	9814	13833	12455	18370	22677	1.40955773

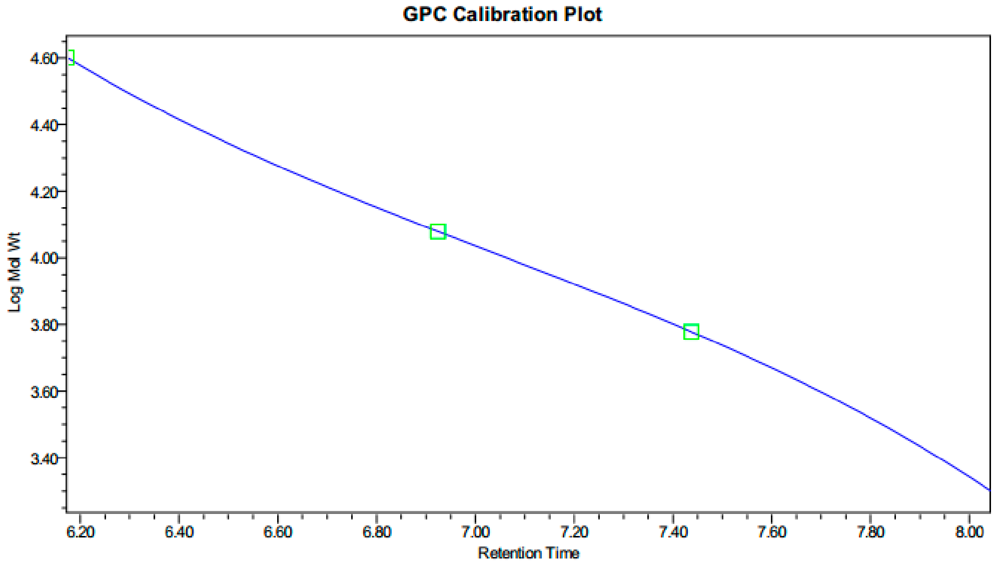

GPC Calibration Table

	Mol Wt (Daltons)	RT (min)	Calculated Weight (Daltons)	% Residual
1	40000	6.172	40000	0.000
2	12000	6.924	12000	0.000
3	6000	7.437	6000	0.000
4	2000	8.042	2000	0.000

Figure S1. GPC of sericin-dextran conjugate (SDC).

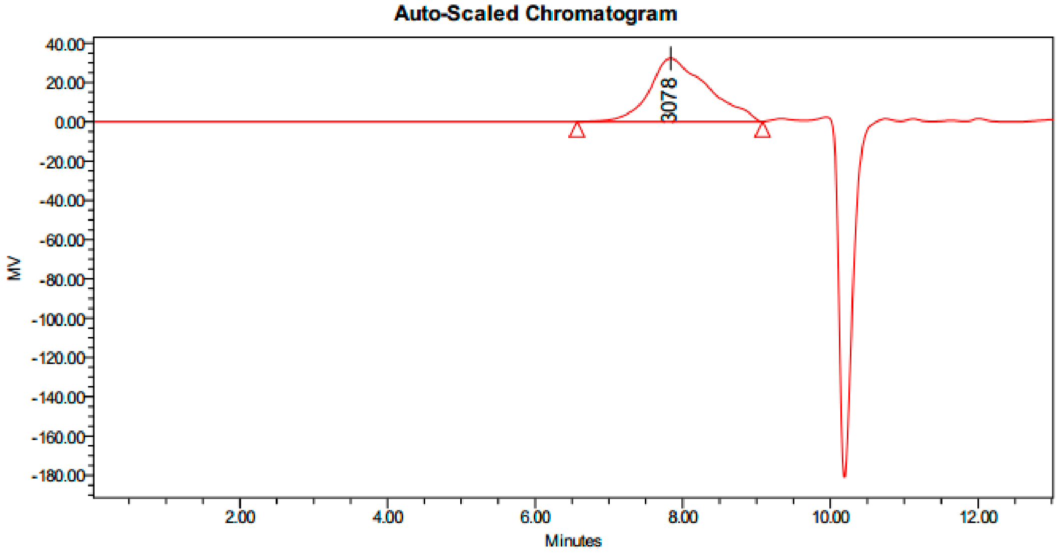

GPC Results

	Retention Time (min)	Area (䃼*sec)	% Height	% Area	Mn	Mw	MP	Mz	Mz+1	Polydispersity
1	7.837	1771314	100.00	100.00	3440	3967	3078	4731	5797	1.15323764

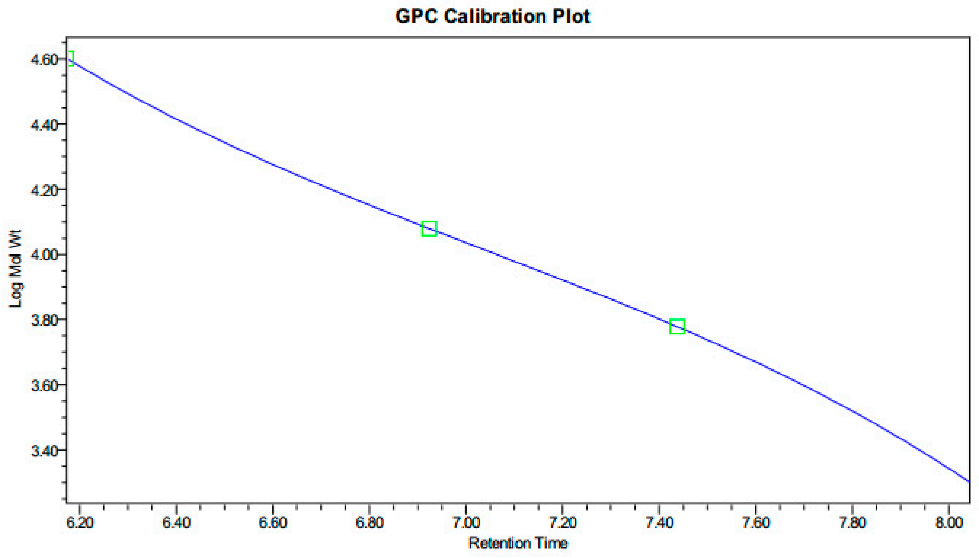

GPC Calibration Table

	Mol Wt (Daltons)	RT (min)	Calculated Weight (Daltons)	% Residual
1	40000	6.172	40000	0.000
2	12000	6.924	12000	0.000
3	6000	7.437	6000	0.000
4	2000	8.042	2000	0.000

Figure S2. GPC of sericin.

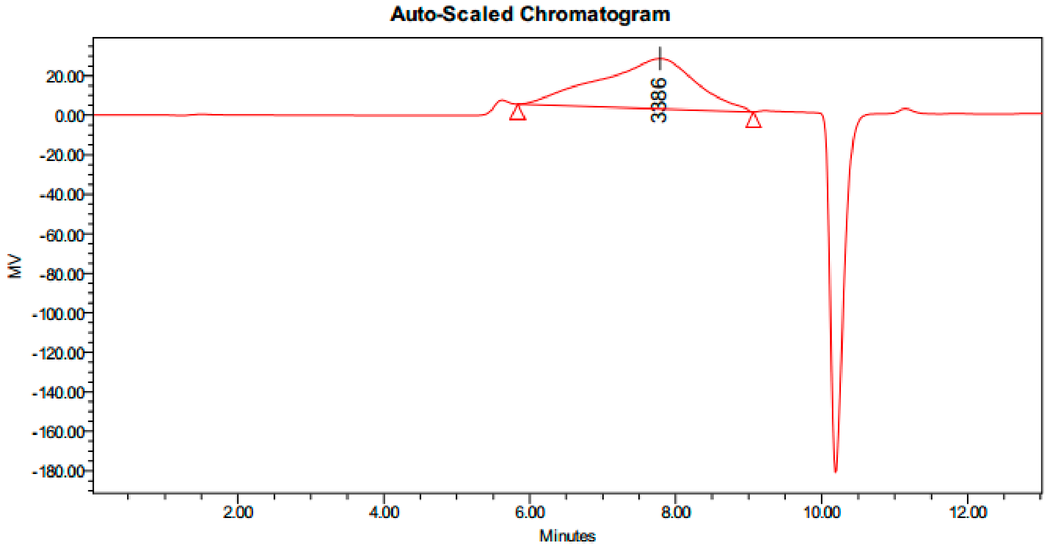

GPC Results

	Retention Time (min)	Area (礦*sec)	% Height	% Area	Mn	Mw	MP	Mz	Mz+1	Polydispersity
1	7.787	2331239	100.00	100.00	5266	8734	3386	14549	20668	1.65847891

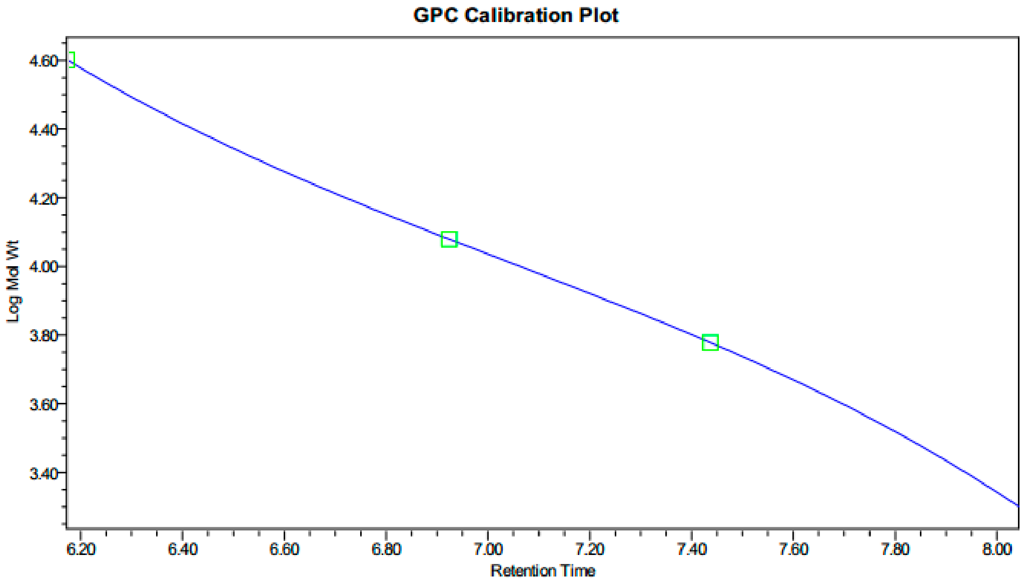

GPC Calibration Table

	Mol Wt (Daltons)	RT (min)	Calculated Weight (Daltons)	% Residual
1	40000	6.172	40000	0.000
2	12000	6.924	12000	0.000
3	6000	7.437	6000	0.000
4	2000	8.042	2000	0.000

Figure S3. GPC of dextran.
